# Aripiprazole Long-Acting Injection During First Episode Schizophrenia—An Exploratory Analysis

**DOI:** 10.3389/fpsyt.2019.00935

**Published:** 2020-01-08

**Authors:** Gloria Giordano, Lorenzo Tomassini, Ilaria Cuomo, Emanuela Amici, Filippo Perrini, Gemma Callovini, Alfonso Carannante, Georgios D. Kotzalidis, Sergio De Filippis

**Affiliations:** ^1^ Villa Von Siebenthal Neuropsychiatric Clinic, Rome, Italy; ^2^ Department of System Medicine, Tor Vergata Hospital, Rome, Italy; ^3^ ASL Roma 1, Istituto Penitenziario Regina Coeli, Rome, Italy; ^4^ Istituto A.T. Beck-Diagnostic Centre, Research and Training in Cognitive-Behavioral Psychotherapy, Rome, Italy; ^5^ NESMOS Department Neurosciences-Mental Health- and Sensory Organs, School of Medicine and Psychology, Sant’Andrea Hospital, Sapienza University of Rome, Rome, Italy

**Keywords:** schizophrenia, first episode psychosis, positive symptoms, negative symptoms, long-acting injectable antipsychotics, aripiprazole, quality of life, personal and social performance

## Abstract

**Background:** Long-acting injectable (LAI) aripiprazole was found to be efficacious in schizophrenia. In common clinical practice, the use of LAIs is often restricted to chronic patients with frequent relapses and poor adherence. Recently, some investigators advanced the idea of early LAI use also in young people with schizophrenia at their first psychotic episode (FEP).

**Objective:** Our study aimed to assess the effect of LAI aripiprazole once monthly (AOM) in the treatment of FEP in patients aged 18–26 years.

**Methods:** We included 50 patients with DSM-5 schizophrenia as assessed with SCID, and used the Clinical Global Impressions Scale-Severity of Illness (CGI-S) and the Positive and Negative Syndrome Scale (PANSS) to assess symptom severity and the World Health Organization Quality of Life (WHOQOL), the Short Form Health Survey (SF-36) and the Personal and Social Performance Scale (PSP) to assess quality of life (QoL) and global health perception at baseline and 3, 6, 9, and 12 months after the first AOM injection.

**Results:** AOM was associated with a progressive improvement, compared to baseline, of both positive (*p* < 0.001) and negative (*p* < 0.001) symptoms and in general psychopathology (*p* < 0.001) and decrease in global severity (*p* < 0.001). We also observed progressive improvement in QoL and social and personal functioning. Treatment adherence was 78% at study endpoint. Our results support that AOM may improve psychotic symptoms, QoL and social functioning in young FEP patients. Further studies should compare AOM to its oral formulation in the treatment of young patients with schizophrenia at the outset of their illness.

## Introduction

Schizophrenia is a psychiatric disorder running a chronic course, often characterized by the alternation of acute and partial remission phases (1, 2). It involves emotional and cognitive impairments (2), and is characterized by positive (hallucinations and delusions, odd and bizarre behavior), negative (e.g., diminished emotional expression, flat affect, alogia, anhedonia, and avolition), as well as cognitive and general symptoms, which are often associated with decline in social and general functioning (3). The onset of the first psychotic episode (FEP) frequently occurs during late adolescence and early adulthood, a sensitive life period, when the pursuit of major life goals may be unexpectedly hampered by the development of major impairments (4). Early onset schizophrenia-spectrum disorders are defined as those with an onset between 13 and 18 years of age (5, 6). About 4–5% of patients diagnosed with schizophrenia had their onset in the above range (5, 6). FEP onset can be gradual or abrupt (4). When gradual, young adults may present with slow and progressive social isolation, deterioration in functioning, and development of positive symptoms over several months or years (1, 7, 8). Despite the possible lack of clear psychotic symptoms (9), often young adults may show a change in self experience, associated with a progressive decline in vocational activities and decreased professional or academic performance (10, 11). With acute onset, abrupt social isolation and disengagement from work and school may arise simultaneously with delusions and hallucinations (10–15) and one symptom may trigger the other or constitute a milieu into which other symptoms may be embedded (16). Abnormal self experience may reset brain activity, thus mediating the onset of positive symptoms and the first psychotic outbreak (17).

Substance use disorder (SUD) is very common among young people with psychosis (18). It may precede or follow the onset of psychotic symptoms or the onset of both may coincide (19–21). Patients with SUD often show worse pre-morbid adjustment, an earlier schizophrenic onset (21), and worse psychotic symptoms than patients without SUD (22). Schizophrenia-SUD comorbidity decreases treatment adherence (23, 24), worsens psychosis outcomes (25), and increases the odds for relapse (26–28), rehospitalisation (27), and suicide attempt (29). The relationship between substance use and treatment non-adherence may be bidirectional, in that one increases the risk for the other (30).

Intervention should not be delayed beyond a 2–3 year critical period after FEP onset, lest long-term prognosis deteriorate (31). Better functional recovery is expected in FEP patients when they show a minimum of three-month sustained remission of both positive and negative symptoms over the first two years of treatment (32). Antipsychotic treatment results in a good response soon after the first episode, but are associated with low clinical and social recovery rates (around 13.5%) (33). This is due to various factors, as poor treatment adherence tends to establish in most patients in the long run [even with long-acting injectable antipsychotic drugs (LAIs), premature treatment termination is more than 50% (34)] and, even when adherence is ensured, after long-term treatment, 15% of patients will still show symptoms of chronic illness (35). Clearly, the management of illness requires constant care for symptom control, relapse prevention, and psychosocial long-term rehabilitation (3), but mainly focuses on drug treatment, mostly antipsychotic drugs (36, 37). Antipsychotic drugs generally act by blocking dopamine D_2_ receptors (38) to acutely control positive and negative symptoms and decrease the frequency and severity of relapses during maintenance treatment (37). Antipsychotics, despite being chemically heterogeneous, are subdivided for convenience in first (or typical) (FGAs) and second (or atypical) generation (SGAs), with the purported distinguishing features but price being unreliable. While the former are usually effective in reducing positive symptoms, but display more severe extrapyramidal adverse effects, the latter were held to be more helpful also for negative symptoms, despite being somewhat detrimental towards metabolism and cardiovascular function, but were also shown as a class not to be better than FGAs in a meta-analysis, even for negative symptoms (39). However, SGAs are a heterogeneous class of drugs and their adverse effects differ greatly from one another (39).

One of the more frequent causes of relapse in schizophrenia is non-adherence (40). While the use of LAIs is often restricted to chronic patients with frequent relapses and poor adherence, some studies showed that LAIs may be effective also at the outset of schizophrenia (41, 42) and there is a strong movement towards implementing LAI treatment early in the course of schizophrenia or other psychoses (43–46). Furthermore, early treatment of FEP co-occurring with SUD was shown to benefit even patients with poor prognosis (47). It should be stressed once again that SUD negatively affects treatment adherence in FEP patients (48). Combining the aforementioned data, we felt that it could be worthwhile to try aripiprazole once-monthly (AOM) in patients at their FEP, comorbid or not with SUD. Our study aimed to assess the effect of AOM in the treatment of young adult FEP patients with or without comorbid SUD, to establish differences between the SUD and the non-SUD FEP groups in terms of changes in clinical symptomatology and other important everyday functioning areas like quality of life (QoL), general health, and personal and social performance.

## Materials and Methods

The study population was composed of 50 FEP inpatients, aged 18–26 years, who met Diagnostic and Statistical Manual of Mental Disorders, 5^th^ edition (DSM-5) criteria for schizophrenia with or without a SUD comorbidity (49), who started treatment with aripiprazole LAI. Efficacy measures included the Clinical Global Impressions Scale-Severity of Illness (CGI-S) (50) and the Positive And Negative Syndrome Scale (PANSS) (51). These assessments were conducted at baseline, and then 3, 6, 9, and 12 months after baseline. Safety and tolerability were assessed through clinical interview, vital signs and laboratory values. The World Health Organization Quality of Life, Brief version (WHOQOL-BREF) (52), the Short-Form Health Survey (SF-36) (53), and the Personal and Social Performance Scale (PSP) (54) were used to evaluate QoL and global health perception at baseline and after 3, 6, 9, and 12 months. Baseline was considered the initiation of LAI treatment that would take place after an oral 28-day aripiprazole run-in phase.

Patients were interviewed with the Structured Clinical Interview for DSM-5 (SCID-5) (55) and diagnosed as having one of the aforementioned disorders. After meeting inclusion criteria, they were explained study aims and methods and provided free, informed consent. The study received approval of the local ethical committee. Excluded were subjects with chronic medical disease, such as diabetes, collagen, and autoimmune disorders, renal failure, liver failure, and severe cardiovascular disorders, major neurological disorders, and incapacity to provide consent.

Patients received monthly intramuscular injections of 400 mg AOM. Injections were conducted in the gluteal muscle. Participants were invited to return each month for their injection; every three months they were subjected to interviews and testing.

Adverse events were monitored if they appeared and rated 0 (absent), 1 (mild), 2 (moderate), and 3 (severe) according to a Likert scale for each event. At every return visit, patients were assessed with the Barnes Akathisia Scale, routine blood chemistry testing, hepatic enzymes, serum prolactin, blood thyroid hormones, vital signs, and electrocardiogram.

### Statistical Analysis

We analysed the sample as intention-to-treat, dealing with missing data with the conservative last observation carried forward (LOCF) method. Three-way ANOVA for between-subject factors (presence vs. absence of SUD) and age-at-onset range [age 18–21 (younger) vs. age 22–26 (older)] and five-level repeated-measures factor time (baseline and 3, 6, 9, 12, months) were performed for each considered measure. Statistical analysis was carried out with the SPSS 25 software (IBM Corporation, Armonk, NY, USA). The statistical significance cutoff was set at *p* < 0.05.

## Results

### Descriptive Statistics

The final sample consisted of 50 Caucasian patients diagnosed with schizophrenia, 39 men (78%) and 11 women (22%). Eighteen patients (36%) had no SUD comorbidity, while 32 (64%) had comorbid SUD. The latter group was composed of 24 men and 8 women; in the comorbid SUD sample, most people used cannabis (N = 21, of whom 15 cannabis alone, 3 with MDMA, 2 with alcohol and 1 with cocaine), while the rest used cocaine (N = 9) or heroin (N = 2) only. The mean age of the entire sample was 23.6 years ± 2.8; 14 patients (28%) belonged to the 18–21 age-at-onset range and 36 (72%) to the 22–26 age-at-onset range. Eleven patients dropped-out before the 12-month follow-up (7 for safety/inefficacy, 2 switched to another LAI, 1 moved to another city, and 1 discontinued due to unbearable akathisia); hence, the final analysis was conducted on 39 participants. However, we kept drop-out cases in our ITT analysis.

### Effects of AOM on PANSS and CGI-S Scores

The ITT analysis with LOCF was used with mixed model ANOVAs involving three independent variables, i.e., SUD (presence/absence) and age-at-onset (18–21/22–26) as between-subjects variables, and time (baseline and 3, 6, 9, and 12 months) as within-subjects variable, and PANSS and CGI-S scores as dependent variables (**Table 1**). **Tables 1** and **3**–**5** show estimated marginal means of the timepoint per timepoint comparisons and the relative significance.

**Table 1:** PANSS and CGI-S, within-subjects pairwise comparisons; significance between successive time-points; *p < 0.05; **p < 0.01; ***p < 0.001.

	Estimated Marginal Means ± SE	Estimated Lower – Upper Bounds	Pairwise Comparison *(p)*
**PANSS positive**			
Baseline	24.31±1.34	21.60 – 27.03	
3 months	23.38±1.44	20.47 – 26.30	0.128
6 months	20.98±1.27	18.41 – 23.55	0.001**
9 months	19.20±1.22	16.75 – 21.66	0.003**
12 months	16.95±1.18	14.55 – 19.34	<0.001***
**PANSS negative**			
Baseline	30.17±1.56	27.02 – 33.32	
3 months	27.86±1.58	24.66 – 31.06	<0.001***
6 months	24.65±1.34	21.92 – 27.35	<0.001***
9 months	22.42±1.26	19.81 – 25.03	<0.001***
12 months	19.74±1.26	17.19 – 22.30	<0.001***
**PANSS general psychopathology**			
Baseline	64.89±2.57	59.70 – 70.07	
3 months	59.37±2.92	53.48 – 65.26	<0.001***
6 months	54.11±2.70	48.67 – 59.56	<0.001***
9 months	50.11±2.66	44.74 – 55.48	<0.001***
12 months	45.81±2.70	40.37 – 51.25	<0.001***
**CGI-S**			
Baseline	5.81±0.11	5.59 – 6.04	
3 months	5.47±0.13	5.20 – 5.73	0.001**
6 months	4.91±0.14	4.62 – 4.90	<0.001***
9 months	4.58±0.16	4.25 – 4.90	0.003**
12 months	4.20±0.21	3.77 – 4.62	p = 0.001**

CGI-S, Clinical Global Impressions Scale-Severity; PANSS, Positive And Negative Syndrome Scale; SE, standard error.

**Figure 1:**
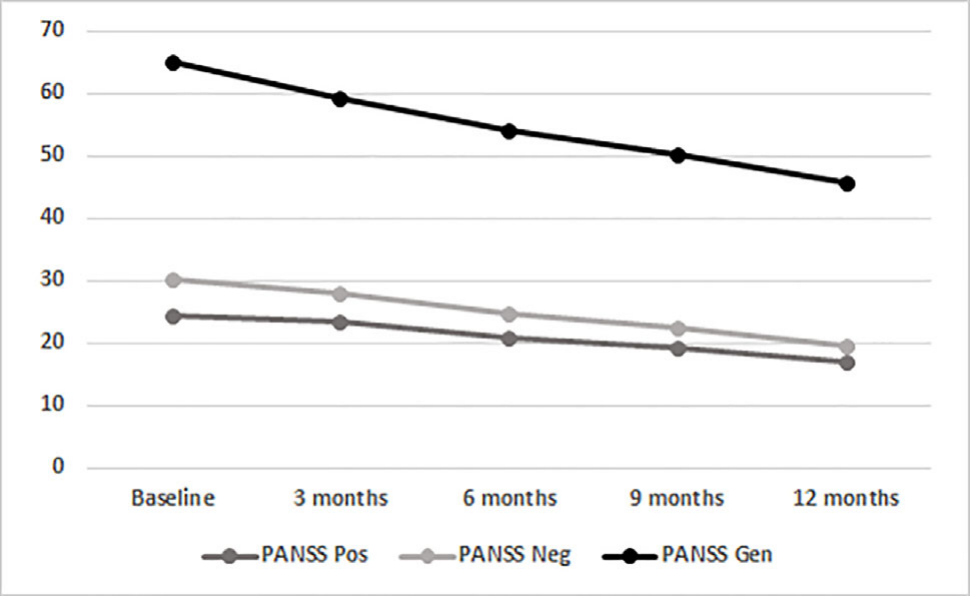
Time course of total PANSS scores in the entire population (BL, baseline; mo, months; PANSS, Positive And Negative Syndrome Scale; Gen, General Psychopathology scale; Neg, Negative scale; Pos, Positive Scale).

LAI aripiprazole treatment was associated with a progressive improvement of positive PANSS scores (from 24.31 at baseline to 16.95 after 12 months; F_(1.696,78.000)_ = 22.946, p < 0.001; **Figure 1**). No main effects of SUD and age-at-onset between variables and interaction effects were observable, indicating no differences in AOM treatment effects between patients with and without a SUD diagnosis and patients with different ages of psychotic onset. Concerning PANSS Negative scores, a main effect of the within-subjects variable time was found (from 30.17 at baseline to 19.74 after 12 months; F_(1.534,__70.547)_ = 40.313; p < 0.001; **Figure 1**; **Table 2**), showing a significant gradual improvement of negative symptoms. Significant main effects of between-subjects variables age-at-onset younger/older (F_(1,__46)_ = 4.725; p = 0.035) and SUD presence/absence (F_(1,__46)_ = 7.878; p = 0.007) were also found, indicating that patients with a younger age of psychotic onset or without a SUD diagnosis were those with more negative symptoms. Furthermore, a significant interaction effect age-at-onset × SUD presence/absence (F_(1,__46)_ = 4.993; p = 0.030) was found, meaning that the highest levels of negative symptoms were found in those patients with an age at onset of psychosis between 18 and 21 years without SUD. Results also showed a significant main effect of within-subjects variable time on the PANSS General Psychopathology scale scores (from 64.89 at baseline to 45.81 after 12 months; F_(1.484,__68.258)_ = 38.572; p < 0.001; **Figure 1**). A significant main effect of the between-subjects variable age-at-onset younger/older (F_(1,__46)_ = 7.343; p = 0.009) was found, indicating that patients with a lower age of psychotic onset were those with more general psychopathology symptoms. No other significant effects were found.

**Table 2:** Within-group main effects of time, Mauchly’s Tests of Sphericity are statistically significant.

	*F* (df)	*p*
**PANSS Positive**	22.946 *_(_*_1.696,78.000)_ *	<0.001
Negative	40.313 _(1.534,70.547)_ *	<0.001
General Psychopathology	38.572 _(1.484,68.258)_ *	<0.001
**CGI-S**	35.661 _(2.100,96.621)_ **	<0.001
**PSP**		
Disturbing and aggressive behavior	33.528 _(1.269,58.353)_ *	<0.001
Personal and social relationships	49.898 _(1.318,60.619)_ *	<0.001
Socially useful activities	53.504 _(1.242,57.112)_ *	<0.001
Self-care	44.071 _(1.338,61.556)_ *	<0.001
**SF-36**		
Social activities	21.742 _(2.517,__115.786)_ *	<0.001
Physical pain	8.167 _(2.499,114.963)_ *	<0.001
Physical health	13.749 _(2.521,115.964)_ **	<0.001
Emotional state	18.859 _(2.517,115.769)_ *	<0.001
General health	9.882 _(2.629,120.941)_ *	<0.001
Mental health	17.129 _(2.354,108.288)_ *	<0.001
Physical activity	7.344 _(1.771,79.695)_ **	0.001
Vitality	15.885 _(2.681,123.333)_ *	<0.001
**WHOQOL-BREF**		
Environment	10.417 _(1.991,91.597)_ **	<0.001
Physical health	14.261 _(1.977,90.947)_ **	<0.001
Psychological	11.892 _(1.525,70.173)_ **	<0.001
Social relationships	8.995 _(2.658,122.281)_ **	<0.001

CGI-S, Clinical Global Impressions Scale-Severity; df, degrees of freedom; PANSS, Positive And Negative Syndrome Scale; PSP, Personal and Social Performance scale; SF-36, Short Form Health Survey; WHOQOL-BREF, World Health Organization Quality Of Life scale-Brief. *Greenhouse-Geisser correction; **Hyun-Feldt correction F.

**Figure 2:**
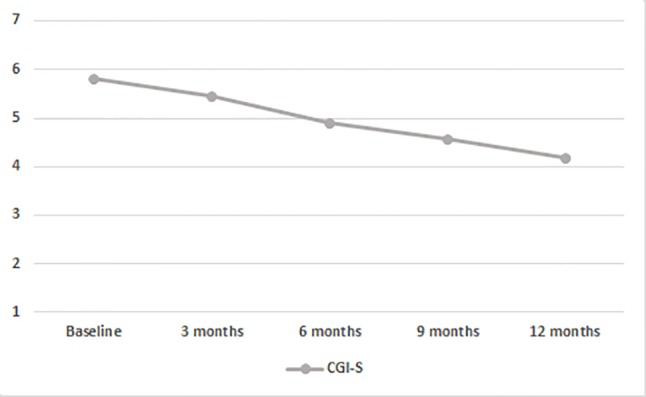
Time course of CGI-S scores in the entire population. BL, baseline; CGI-S, Clinical Global Impressions Scale-Severity of Illness.

Mixed model ANOVAs with CGI-S scores showed a significant steady decrease of global severity (from 5.81 at baseline to 4.20 after 12 months; F_(2.100,96.621)_ = 35.661; p < 0.001; **Figure 2**; **Table 2**). No significant between-subjects effects were found, but in one instance, the age younger/older variable approached significance (F_(1,46)_ = 3.836; p = 0.056), suggesting that patients with age at onset of psychosis between 18 and 21 years scored higher on the CGI-S.

### AOM Effects on Global Health Perception and Treatment Adherence

We observed a constant increase in QoL, as shown by scores on selected WHOQOL and SF-36 domains, and of social and personal functioning, on all PSP subscales (**Tables 3**–**5**). WHOQOL, SF-36, and PSP scores showed within-subjects effects of time on every subscale. Significant between-subjects main effects of age younger/older were observed on the physical (F_(1,46)_ = 5.396; p = 0.025), psychological (F_(1,46)_ = 7.956; p = 0.007) and environment (F_(1,46)_ = 6.733; p = 0.013) WHOQOL domains and on general health perceptions (F_(1,46)_ = 4.106; p = 0.049) SF-36 subscale, indicating worse QoL and social and personal functioning in patients with a psychotic onset between 18 and 21 years (**Table 3**). No other significant effects emerged.

**Table 3:** WHOQOL-BREF within-subjects pairwise comparisons; significance between successive time-points;*p < 0.05; **p < 0.01; ***p < 0.001.

	Estimated Marginal Means ± SE	Estimated Lower – Upper Bounds	Pairwise Comparison *(p)*
**WHOQOL – Environment**			
Baseline	54.93±4.23	46.41 – 63.46	
3 months	63.17±4.32	54.47 – 71.87	p < 0.001***
6 months	63.68±3.30	57.03 – 70.33	0.797
9 months	65.78±2.89	59.95 – 71.61	0.078
12 months	68.03±3.03	61.92 – 74.13	0.132
**WHOQOL – Physical Health**			
Baseline	58.19±4.77	48.57 – 67.81	
3 months	62.37±4.51	53.29 – 71.45	0.024*
6 months	65.30±4.02	57.19 – 73.40	0.047*
9 months	70.46±3.58	63.25 – 77.67	<0.001***
12 months	72.76±3.40	65.91 – 79.61	0.097
WHOQOL – Psychological			
Baseline	54.52±5.17	44.10 – 64.93	
3 months	55.30±4.70	45.83 – 64.77	0.699
6 months	60.16±3.83	52.44 – 67.87	0.007**
9 months	65.85±3.44	58.91 – 72.79	<0.001***
12 months	68.84±3.16	62.47 – 75.20	0.029*
**WHOQOL – Social Relationships**			
Baseline	52.91±4.69	43.45 – 62.37	
3 months	57.00±4.47	48.00 – 66.00	0.124
6 months	59.54±4.36	50.76 – 68.32	0.176
9 months	64.21±4.10	55.96 – 72.47	0.078
12 months	68.15±3.83	60.42 – 75.87	0.074

SE, standard error; WHOQOL-BREF, World Health Organization Quality Of Life scale-Brief.

**Table 4:** SF-36 within-subjects pairwise comparisons; significance between successive time-points; *p < 0.05; **p < 0.01; ***p < 0.001.

	Estimated Marginal Means ± SE	Estimated Lower – Upper Bounds	Pairwise Comparison *(p)*
**SF – Social activities**			
Baseline	33.54±4.23	25.02 – 42.05	
3 months	41.11±4.29	32.47 – 49.75	0.021*
6 months	46.67±3.32	39.97 – 53.37	0.015*
9 months	53.01±3.23	46.50 – 59.53	<0.001***
12 months	56.28±3.11	50.02 – 62.54	0.074
**SF – Physical pain**			
Baseline	41.93±5.15	31.55 – 52.32	
3 months	40.47±4.85	30.70 – 50.24	0.544
6 months	46.43±5.55	35.26 – 57.61	0.051
9 months	50.22±4.83	40.48 – 59.96	0.041*
12 months	56.99±4.26	48.41 – 65.56	0.051
**SF – Physical health**			
Baseline	28.77±5.08	18.54 – 38.99	
3 months	36.93±5.94	24.97 – 48.89	0.068
6 months	43.80±5.19	33.34 – 54.26	0.021*
9 months	46.86±5.19	36.40 – 57.32	0.115
12 months	55.86±4.95	45.88 – 65.83	0.001**
**SF – Emotional state**			
Baseline	18.41±5.70	6.96 – 29.90	
3 months	21.15±6.16	8.75 – 33.55	0.359
6 months	34.85±5.75	23.26 – 46.43	0.001*
9 months	40.70±5.46	29.69 – 51.70	0.057
12 months	47.75±5.38	36.92 – 58.58	0.017*
**SF – General health**			
Baseline	45.50±4.46	36.52 – 54.48	
3 months	46.54±4.25	37.99 – 55.10	0.690
6 months	50.24±3.80	42.58 – 57.90	0.166
9 months	54.84±3.39	48.01 – 61.67	0.038*
12 months	58.62±3.44	51.69 – 65.56	0.101
**SF – Mental health**			
Baseline	41.63±4.40	32.78 – 50.49	
3 months	48.32±4.40	39.44 – 57.19	0.001**
6 months	50.58±3.80	42.93 – 58.23	0.291
9 months	56.00±3.41	49.13 – 62.87	0.001**
12 months	57.85±3.28	51.24 – 64.47	0.224
**SF – Physical activity**			
Baseline	54.17±6.26	41.56 – 66.77	
3 months	53.47±6.21	40.94 – 65.99	0.469
6 months	59.99±5.20	49.51 – 70.47	0.005**
9 months	61.74±4.88	51.92 – 71.57	0.182
12 months	64.65±4.58	55.42 – 73.88	0.223
**SF – Vitality**			
Baseline	38.66±4.23	30.14 – 47.18	
3 months	46.85±4.56	37.66 – 56.04	0.011*
6 months	51.11±4.38	42.29 – 59.94	0.038*
9 months	54.57±4.20	46.10 – 63.04	0.028*
12 months	57.95±4.03	49.84 – 66.07	0.086

SE, standard error; SF-36, Short Form Health Survey.

**Table 5:** PSP within-subjects pairwise comparisons; significance between successive time-points; *p < 0.05; **p < 0.01; ***p < 0.001.

	Estimated Marginal Means ± SE	Estimated Lower – Upper Bounds	Pairwise Comparison *(p)*
**PSP - Disturbing and aggressive behavior**			
Baseline	25.44±2.29	20.82 – 30.07	
3 months	29.03±2.55	23.89 – 34.18	<0.001***
6 months	33.50±2.65	28.16 – 38.85	<0.001***
9 months	39.32±2.71	33.85 – 44.79	<0.001***
12 months	46.21±3.50	39.16 – 53.26	<0.001***
**PSP - Personal and social relationships**			
Baseline	19.95±1.78	16.35 – 23.54	
3 months	23.74±1.86	19.98 – 27.49	<0.001***
6 months	28.54±1.86	24.79 – 32.28	<0.001***
9 months	34.42±1.98	30.43 – 38.41	<0.001***
12 months	41.87±2.78	36.27 – 47.46	<0.001***
**PSP - Socially useful activities**			
Baseline	19.95±1.77	16.37 – 23.52	
3 months	23.32±1.79	19.70 – 26.94	<0.001***
6 months	29.31±1.80	25.67 – 32.95	<0.001***
9 months	35.05±1.97	31.07 – 39.03	<0.001***
12 months	42.60±2.87	36.81 – 48.39	<0.001***
**PSP – Self-care**			
Baseline	19.45±1.98	15.48 – 23.48	
3 months	24.47±2.11	20.22 – 28.73	<0.001***
6 months	29.70±2.26	25.14 – 34.25	<0.001***
9 months	34.39±2.34	29.66 – 39.11	<0.001***
12 months	43.32±3.33	36.60 – 50.04	<0.001***

PSP, Personal and Social Performance scale; SE, standard error.

WHOQOL, SF-36, and PSP scores showed within-subjects effects of time on every subscale. Significant between-subjects main effects of age-at-onset were observed on the environment (*F*_(1,35)_ = 5.127; p = 0.30) and psychological (*F*_(1,35)_ = 6.925; p = 0.13) WHOQOL domains, on the emotional role functioning (*F*_(1,__35)_ = 4.260; p = 0.46) and general health perceptions (*F*_(1,35)_ = 4.168; p = 0.49) SF-36 subscales, and on the personal and social relationships (*F*_(1,35)_ = 7.284; p = 0.11) and self-care (*F*_(1,35)_ = 6.425; p = 0.16) PSP subscales, indicating worse QoL and social and personal functioning in patients with a psychotic onset between 18 and 21 years (**Table 2**).

**Figure 3:**
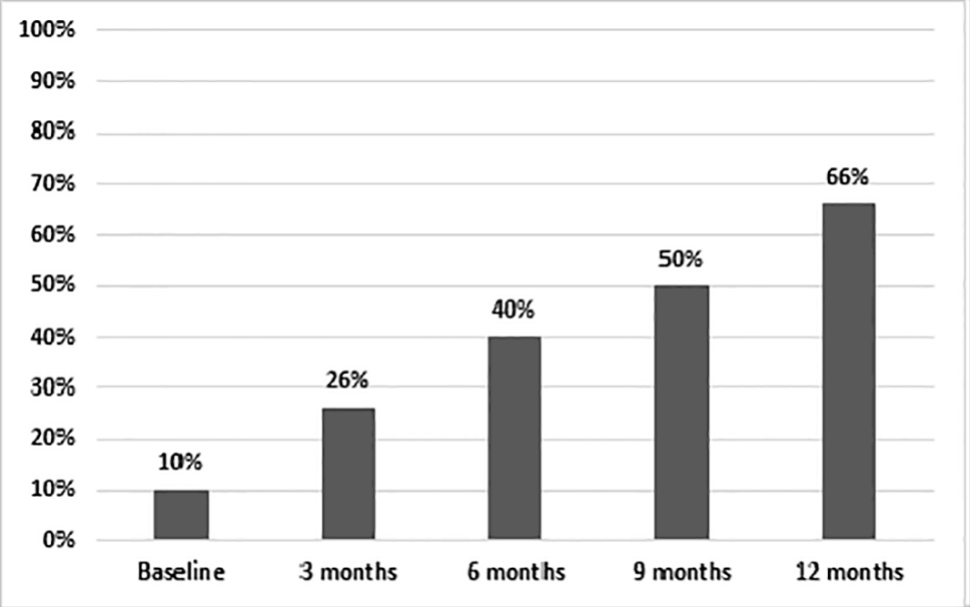
Proportion of patients with <80 PANSS total score.

**Figure 4:**
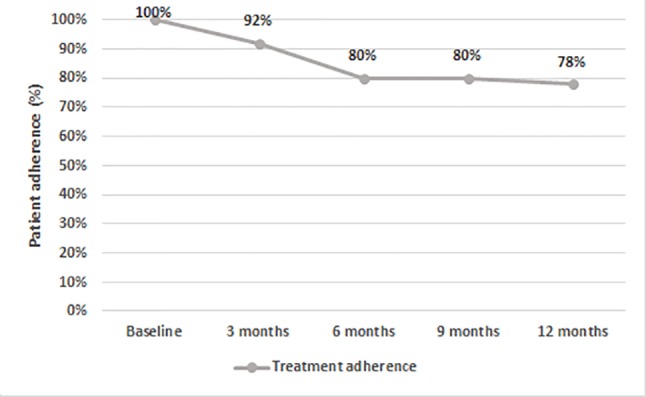
Percentage of patients (N = 50) adherent to treatment throughout the study.

After 12 months, 66% of patients on AOM had a total PANSS score of <80 (**Figure 3**). Treatment adherence was high during the entire study, with a final mean adherence of 78% (**Figure 4**).

### Safety

We did not observe significant side effects in our study, despite close monitoring. One patient developed restless leg syndrome and akathisia, which was rated moderate, but he could not tolerate it and abandoned the study, despite having benefitted from the treatment. No other patient left the study for a side effect or inefficacy, but for their own will. Patients who dropped out for inefficacy/unbearable side effects (N = 7) were removed by the physician.

## Discussion

Our results support that AOM administration for one year may be associated with improvement of schizophrenic symptoms (both positive and negative) and with better QoL and social functioning in young patients with a FEP. Only during recent times, the possibility to manage schizophrenia onset with LAIs right from the start has been investigated (42, 56). Generally, LAI therapy is not considered as first-line for FEP. In clinical practice, LAIs are usually prescribed to frequently relapsing, non-adherent patients who need maintenance treatment (57, 58). However, there is a recent change in therapeutic trends of FEP, with the Texas Medication Algorithm Project (TMAP) for antipsychotics in schizophrenia (59), and the new Canadian guidelines proposing the use of LAI antipsychotics during the entire course of schizophrenia spectrum disorders, including the first 2–5 years (60). In addition, the guidelines for the use and management of LAI antipsychotics in clinical practice by the French Association for Biological Psychiatry and Neuropsychopharmacology (AFPBN) suggest systematic offering of LAI antipsychotics as first line treatment to patients with schizophrenic who need maintenance treatment (61).

Adherence to oral prescriptions is frequently overestimated and non-adherence to treatment is not estimated with precision (40). Recently diagnosed FEP patients may be at-risk for poor adherence (48, 62, 63). Comorbid substance use and poor insight, which may be very frequent in adolescence, contribute to poor adherence in adolescent FEP (64). Partial and incomplete adherence to treatment are identified as strong predictors of relapse in FEP patients (2, 65, 66). After the remission from FEP, the mean one-year risk of symptoms recurrence for treatment discontinuation is 77%, compared to 3% for treatment continuation (58). Recurrence of psychotic symptoms after discontinuing medication occurs within a short period of time, about two weeks, even in patients with a better prognosis, and does not depend on the time to complete remission or the duration of remission itself (35, 67, 68). Symptom relapse may result in impoverishment of social relationships, discontinuation of work or education, stigmatisation, reduced self-esteem and QoL, and may have a negative emotional impact on the patient and his/her family, thus worsening and deteriorating patient’s life in its entirety. In a randomized, double-blind, flexible-dose, multicenter study (48), adherence to oral antipsychotic treatment among patients with first episode of schizophrenic disorders was very low. The study highlighted the importance of treatment response in preventing discontinuation against medical advice and poor adherence to medication in FEP patients. In another study (62) the one-year treatment adherence of 112 FEP patients was less than 75%. Parkinsonian side effects increased the likelihood (hazard ratio = 41.22; 95% CI = 2.30 to 737.89; *p* = 0.01), and better executive function decreased the likelihood (hazard ratio = 0.40; 95% CI = 0.18 to 0.88; *p* = 0.02) that patients discontinued maintenance medication after a first relapse (62). Furthermore, in another study, 68% of 50 patients at an early phase of schizophrenia showed a period of non-adherence; medication non-adherence robustly predicted a return of psychotic symptoms during the early phase of schizophrenia (63). The risk of relapse in schizophrenia increased immediately after interrupting antipsychotic medications and remained high over time (2). Maintaining treatment adherence was found to be strongly associated to a reduced risk of relapses, fewer hospitalizations, and a better QoL (69). A recent review (70) showed that lack of insight, mistrust in the effectiveness of medication, and substance abuse were strongly associated to poor adherence to treatment and greater relapse risk, hospitalisation, and suicide were the most frequent consequences. Titus-Lay and colleagues (71) analysed adherence in 47 FEP patients, subdivided according to three different types of antipsychotic prescription history, i.e., oral only, LAI only, and both. The average proportions of days with medication were 32, 76, and 75% respectively. The authors concluded that LAIs were associated with better adherence compared with oral antipsychotics in patients with early psychosis.

Several advantages of LAIs over oral antipsychotics have been shown in several studies (41, 72–76). In fact, therapeutic contacts could be increased since LAIs are administered by a mental health professional; non-adherence may be easily recognized, increasing the chances of quick intervention; the risk of accidental or deliberate overdose is decreased; drug-drug interactions risks are reduced by avoiding first-pass metabolism; finally injections may stabilise plasma drug concentrations, thus avoiding fluctuations below or above the desired range (77). A recent Canadian naturalistic study (78) showed that LAI-treated FEP patients displayed clinical and functional improvements after 12 months.

Comparisons of oral antipsychotics to LAIs are still inconsistent (60, 79, 80). In a Korean study (80), relapse rates of FEP patients treated with LAI or oral risperidone were compared. At the 1- and 2-year follow-up, patients treated with risperidone LAI showed lower rates of relapses, higher adherence rates (68 vs. 38%), and longer periods of adherence than patients treated with oral risperidone. Furthermore, patients treated with risperidone LAI showed a greater reduction in total PANSS scores (10 vs. 2%; *p* = 0.001), in CGI-S scores (10 vs. 2.5%; *p* = 0.001), and a better functional improvement on the Global Assessment of Functioning (GAF) scale (25% *vs.* 0.5%; *p* = 0.001). Weiden and colleagues (79) compared post-FEP maintenance treatment with risperidone LAI *vs.* oral risperidone. At 12 weeks, patients on risperidone LAI showed a higher rate of adherence to treatment than patients on oral risperidone (89 vs. 59%; *p* = 0.035). The LAI had no additional effect on stigmatization or more side effects than the oral formulation. In the continuation of the study (81), the patients were analysed 104 weeks after treatment initiation. The authors found no significant differences between risperidone LAI and oral formulation in terms of adherence or attitude towards medication, a measure of insight.

In a more recent randomized controlled trial (45), patients in their early phase of schizophrenia stabilized on risperidone LAI or various oral antipsychotic drugs, i.e., risperidone, olanzapine, or quetiapine, showed no differences in CGI-S and PANSS scores. The subsequent *post hoc* analysis revealed that scores on the PANSS Negative scale decreased for both groups, and during the stabilisation phase this decrease was greater and only significant for those patients treated with risperidone LAI. However, intriguingly, from stabilisation to study-end, only the oral group showed a significant decrease in negative symptom scores (*p* = 0.005).

An observational retrospective study (82) focused on the use of second-generation LAIs in adolescence. The use of risperidone (36.7%), aripiprazole (40%), and paliperidone palmitate (23.3%) LAI was mainly based on a history of low compliance (90%) and/or poor insight (73.3%). A mean improvement of 31.7 (SD = 8.7) between admission and discharge was observed on the Children’s Global Assessment Scale (CGAS); no differences were found between the different LAIs. Although they were generally well tolerated, 23.3% of patients reported mild short-term side effects, which were more frequent with risperidone than with aripiprazole (*p* = 0.014) (82).

### Other Issues

In this study we observed positive effects on aggression on the PSP-Disturbing and aggressive behavior domain (**Table 5**). Scores on this domain tend to be high at an early stage in FEP patients, in which intense agitation, combined with positive psychotic symptoms may ensue in aggression. Acute treatment often requires sedation until psychosis subsides. While LAI treatment at this stage is not usually possible to enforce, we have developed in our hospital a treatment guideline that allows an acutely FEP patient to reach a LAI treatment with no delay (**Table 6**).

**Table 6:** Intramuscular and oral aripiprazole administration to acutely agitated patients with psychosis.

Timing	9.75 mg IM	Oral 10 mg	LAI 400 mg
Days 0–5			
Morning	×		
Evening	×		
Bedtime	×		
Days 6–9			
Morning	×		
Evening		×	
Bedtime	×		
Days 10–12			
Morning	×		
Evening		×	
Bedtime		×	
Day 14			
Morning			×
Evening		×	
Bedtime		×	
Days 15–22			
Morning			
Evening		×	
Bedtime		×	
Days 23–29			
Morning			
Evening		×	
Bedtime		×	
Days 20–30 to 2^nd^ LAI 400 mg administration			
All day long			

Strongly agitated patients are prescribed intramuscular delorazepam 2–5 mg or oral lorazepam 4 mg, q 8–12 h. Such procedure allows us to reduce prior drug treatment by 25% each week. × = administration.

Briefly, we ensure a calming effect with 9.75 mg IM aripiprazole injections up to three times daily until the patient gains insight into his/her need to assume this medication and provide consent for LAI treatment, and establish oral treatment with 10 mg oral aripiprazole twice daily for one month, while administering 400 mg AOM on day 14, after having him/her stabilized with oral and IM medication. After the first month, the patient is discharged and AOM is administered monthly thereafter. In fact, we found repeated administration of 9.75 aripiprazole IM to be effective in treating acute psychomotor agitation in acutely psychotic patients (83); this formulation has shown anti-aggressive effects in psychosis-elicited aggression, although the quality of evidence of the studies considered in this systematic review was quite poor (84).

Here we found significant interaction effects for age × presence/absence of SUD on negative symptoms, in that earlier onset (18–21 years) patients without SUD had more severe negative symptoms and a between-subjects interaction of age × presence/absence of SUD on global severity, which was higher in the same group. This matches literature results, in that late-onset women with schizophrenia were shown to have less severe negative and more positive symptoms (85), and patients with comorbid SUD had fewer negative and more positive symptoms (86) and a strong association between cannabis abuse and fewer negative symptoms has been confirmed for cannabis users (87). It should be recalled that more than half of our sample used cannabinoids as recreational substances.

Summarizing our exploratory analysis, we found LAI aripiprazole in FEP to be paralleled by reductions in all PANSS subscale and CGI-S scores at each time point during a 1-year follow-up, compared to baseline, as well QoL improvement in some domains. Our study indicates the possible use of a LAI in a young population with schizophrenia with or without comorbid SUD and a remarkably low occurrence of adverse events.

### Limitations

The small sample size, the open-label design, and the absence of a control group prevent us from drawing firm conclusions. Furthermore, to address missing data, we used the LOCF procedure, which has been reported to have some fallacies, and may not be as conservative as it was previously held, but is adequate in randomized longitudinal studies (88). Since this was an exploratory study, we did not correct for multiple comparisons, since the Bonferroni would have been too conservative. Replication is needed in a larger sample and with a longitudinal, prospective design to better understand the role of LAIs in FEP management. Furthermore, there is need to compare FEP patients with people affected by chronic schizophrenia-spectrum disorders to clarify whether there are differences in the response to LAI treatment and to dispel the myth that we should wait for non-adherence to develop before treating a young person with a LAI. The LAI *vs.* oral antipsychotic comparison is another issue to address adequately and will require head-to-head comparisons. More studies are needed to better understand the role of LAIs in overcoming non-adherence in people with schizophrenia.

## Conclusions

The results of our study supports the hypothesis that LAI aripiprazole may improve psychotic symptoms, QoL and social functioning in young patients with FEP. Further studies should focus on comparing the long-acting injectable to the oral formulation of aripiprazole in the treatment of young patients with schizophrenia at the outset of their illness.

## Data Availability Statement

The datasets generated for this study are available on request to the corresponding author.

## Ethics Statement

The studies involving human participants were reviewed and approved by Villa von Siebenthal Ethical Committee. The patients/participants provided their written informed consent to participate in this study.

## Author Contributions

GG and SF designed the study and wrote substantial portions of the introduction and discussion. LT, IC, GC, and AC collected data and introduced them in the database. EA created the database and introduced data. FP performed statistical analyses and wrote the *Results* section with EA and GK. GK, GG, IC, EA, GC, and AC drafted the first version and wrote the paper. FP, SF, and GK supervised the final version.

## Conflict of Interest

The authors declare that the research was conducted in the absence of any commercial or financial relationships that could be construed as a potential conflict of interest.
